# Methicillin-Resistant *Staphylococcus aureus* Colonization: A Three-Year Prospective Study in a Neonatal Intensive Care Unit in Italy

**DOI:** 10.1371/journal.pone.0087760

**Published:** 2014-02-05

**Authors:** Daniela M. Geraci, Mario Giuffrè, Celestino Bonura, Domenica Matranga, Aurora Aleo, Laura Saporito, Giovanni Corsello, Anders Rhod Larsen, Caterina Mammina

**Affiliations:** 1 Department of Sciences for Health Promotion and Mother-Child Care “G. D’Alessandro”, University of Palermo, Palermo, Italy; 2 PhD School in Food and Human Nutrition, University of Palermo, Palermo, Italy; 3 Postgraduate Specialty School of Hygiene and Preventive Medicine, University of Palermo, Palermo, Italy; 4 Statens Serum Institute, Copenhagen, Denmark; Columbia University, United States of America

## Abstract

**Background:**

Methicillin resistant *Staphylococcus aureus* (MRSA) is a major etiological agent of infection in neonatal intensive care units (NICUs). Routes of entry of this organism can be different and the transmission pathway complex. Colonized neonates are the main endogenous reservoir.

**Methods and Results:**

We conducted a prospective three-year study on MRSA colonization recruiting 722 neonates admitted between 2009 and 2012. Nasal swabs were cultured weekly and MRSA isolates were submitted to molecular typing. The annual incidence density of acquisition of MRSA ranged from a maximum of 20.2 cases for 1000 patient-days during the first year to a minimum of 8.8 cases in the second one to raise again up to 13.1 cases during the third year. The mean weekly colonization pressure fluctuated from 19.1% in the first year to 13.4% in the second year and 16.8% in the third year. It significantly correlated with the number of MRSA acquisitions in the following week. Overall, 187 (25.9%) subjects tested positive for MRSA. A non multiresistant, *tst* positive, ST22-MRSA-IVa *spa* t223 strain proved to be endemic in the NICU, being identified in 166 (88.8%) out of 187 colonized neonates. Sporadic or epidemic occurrence of other strains was detected.

**Conclusions:**

An MRSA strain belonging to the *tst*1 positive, UK-EMRSA-15/ “Middle Eastern Variant” appeared to be endemic in the NICU under investigation. During the three-year period, substantial changes occurred in case-mix of patients moving towards a higher susceptibility to MRSA colonization. The infection control procedures were able to decrease the colonization rate from more than 40% to approximately 10%, except for an outbreak due to a CA-MRSA strain, ST1-MRSA-IVa, and a transient increase in the colonization prevalence rate coincident with a period of substantial overcrowding of the ward. Active surveillance and molecular typing contributed to obtain a reliable picture of the MRSA dissemination in NICU.

## Introduction

Methicillin resistant *Staphylococcus aureus* (MRSA) is a major etiological agent of infection in neonatal intensive care units (NICUs) [1]. Very low birth weight (VLBW) infants and those with serious underlying diseases, such as malformations and surgical conditions, are generally most vulnerable to MRSA infections [1], [2]. Use of invasive devices, which is very frequent in these patients, is also associated with increased risk for invasive MRSA infections [3]. Routes of entry of this organism in NICU can be different and the transmission pathway complex [2].

Colonized neonates are the main endogenous reservoir of MRSA in NICU, but their relatives and the healthcare workers (HCWs) may all play a role in the transmission chain [2]. Colonization pressure has also been reported as a major variable affecting rates of MRSA acquisition [4]. Overcrowding and understaffing have been proved to promote cross-transmission of MRSA [5].

Clinical cultures are generally thought to underestimate MRSA prevalence in NICU, whereas active surveillance cultures (ASCs) are able to detect MRSA colonization and provide otherwise unavailable information helpful to control and prevent MRSA spread [6].

Despite NICU being a setting where healthcare associated (HA)-MRSA clones have been frequently reported, community associated (CA)-MRSA clones are increasingly described as nosocomial pathogens [7]. Spread of CA-MRSA has added further complexity to epidemiology of MRSA in NICUs as well as to the management and control strategies of MRSA infections [2]. Molecular epidemiology can greatly contribute to address these issues by accurately identifying MRSA clones [8].

The purposes of this study were: a) to assess the prevalence of MRSA colonization in infants admitted to the tertiary NICU of the teaching hospital “P. Giaccone”, Palermo, Italy, in the period June 2009– June 2012; b) to identify and characterize by molecular typing the MRSA strains circulating in the NICU; c) to describe the temporal trend of MRSA colonization in relation to the infection control strategies.

## Methods

### Study Design and Setting

This was a prospective three-year study of MRSA colonization taking place at the tertiary NICU of the teaching hospital “Paolo Giaccone”, Palermo, Italy. The NICU annually admits about 250 infants of all gestational ages. Because it is associated with the regional reference centre for genetic diseases, the NICU has a high prevalence of neonates with malformations (20% approximately), as well as of outborn admissions (35% approximately). Moreover, a further 20% proportion of patients has complex conditions requiring subspecialty medical or surgical care. The NICU includes one intensive care room consisting of 8 cot spaces and one intermediate care room including 8 further cot spaces. The average nurse to patient ratio is 1∶3 and 1∶4 in the two sections, respectively. The NICU ward is open to parents for 2 hours in the morning and 4 hours in the afternoon, so that they can be progressively involved in the general care of their child under the guidance and supervision of the staff. Early breastfeeding is supported.

Inclusion criteria were admission to NICU between June 16, 2009 and June 15, 2012, a hospital stay of at least 48 hours and the collection of at least one nasal swab. Demographic, clinical and microbiological data were prospectively collected. At admission demographic characteristics, gestational age, birth weight, inborn or outborn condition, delivery type, APGAR score and comorbid conditions were recorded. During the NICU stay, qualitative and quantitative data were collected about the following variables: presence of central vascular access devices, endotracheal intubation and mechanical ventilation, nasal continuous positive airway pressure (nCPAP), peripheral catheters, type of feeding (i.e. parenteral nutrition, enteral nutrition with oral suction or gavage, breast milk, formula), eventual need and timing of surgery, antibacterial drug therapy, length of stay and survival status at discharge.

Infants were categorized as colonized by MRSA when at least one nasal swab tested positive.

For each of the 156 weeks of the study, patient-days and patient-days with MRSA were calculated. The mean weekly colonization pressure (WCP) was also calculated, according with Merrer et al. [9], as the ratio between the number of MRSA positive patient-days in each week × 100 and the total number of patient-days in the week.

The study protocol was approved by the Ethics Committee of the Azienda Ospedaliero-Universitaria Policlinico “P. Giaccone”, Palermo, Italy, and informed consent was sought in accordance with the principles of the Declaration of Helsinki. Written informed consent was obtained from the parents or guardians of the neonates.

### Infection Control Strategies

Since June 2009, a surveillance protocol is routinely in place which includes nasal and rectal swabs obtained on a weekly basis from all infants staying in the NICU to monitor the prevalence of carriage with MRSA, multidrug resistant (MDR) Gram negatives and glycopeptide-resistant enterococci. Universal screening at admission was routinely performed at the beginning of the surveillance program, but after six months it was discontinued because of organizational problems and a low rate of positive culture. A policy of appropriate management of invasive devices is well established in the NICU, including removal of central umbilical catheter at 72 h and substitution of any further central venous line within a maximum of 21 days and in any case of suspected/documented blood stream infection.

Measures taken to control MRSA spread in NICU included contact precautions, use of dedicated equipment, periodic training sessions on hand hygiene and intensified sanitation of surfaces in the colonized and infected neonates cot spaces. Moreover, medical and nursing staff paid special attention to avoid overcrowding and relative understaffing, to minimize length of hospital stay and to promote safe use of invasive devices. Colonized and non colonized neonates were divided in two cohorts, but without the possibility of providing dedicated nursing staff. No neonates were treated with mupirocin for decolonization. During the first six months of the study, following the detection of a high prevalence of MRSA colonization among NICU patients, surveillance cultures from HCWs had been also performed showing a carriage prevalence of about 8%. Nasal mupirocin treatment was administered to each colonized HCW and decolonization was confirmed by culture of anterior nares. Colonized staff were not furloughed, because of the possible negative impact of understaffing on infection control procedures.

### Surveillance Cultures

Surveillance specimens from the anterior nares of neonates were obtained with cotton swabs, immediately transferred to the laboratory and processed within 2 hours. Nasal swabs were incubated overnight in Brain Hearth Infusion (BHI) broth (OXOID, Basingstoke, UK) and then plated onto mannitol salt agar (OXOID), incubated in air at 35°C and examined at 24 and 48 h. Presumptive *S. aureus* isolates were identified according to standard methods. MRSA isolates were searched for by colony screening onto oxacillin agar (Mueller-Hinton with oxacillin 6 mg/L). Isolates were confirmed as MRSA by the cefoxitin disk diffusion test and PCR for detection of *mec*A [10].

The first isolate from each MRSA colonized patient was submitted to genotyping and antibiotic susceptibility test. Antibacterial drug susceptibility was routinely performed using the disk diffusion method by determining the susceptibility of each isolate to erythromycin, clindamycin, sulfamethoxazole-trimethoprim, tetracycline, ciprofloxacin, gentamicin, tobramycin, linezolid, rifampicin, vancomycin and teicoplanin. *S. aureus* ATCC 25923 was used as the quality control strain. Inducible resistance to clindamycin was tested by “D test” placing a 15 µg erythromycin disk 15 mm apart edge to edge from a 2 µg clindamycin disk on a Mueller-Hinton agar plate previously inoculated with an MRSA isolate. A D-shape of the zone around clindamycin facing the erythromycin disk indicated inducible clindamycin resistance. Results were interpreted using the European Committee on Antimicrobial Susceptibility Testing (EUCAST) clinical breakpoints (http://www.eucast.org/fileadmin/src/media/PDFs/EUCAST_files/Breakpoint_tables/).

### Molecular Typing

Staphylococcal cassette chromosome (SCC) *mec* was typed by the multiplex PCR method described by Milheiriço et al. [11]. Genotypic characterization of the MRSA isolates was performed by multilocus variable number tandem repeat fingerprinting (MLVF) [12]. Gel images were stored as TIFF files. Banding patterns were analyzed both visually and by using Bionumerics version 5.10 (Applied Maths, Sint-Martens-Latem, Belgium).

The *S. aureus* Genotyping Kit (Alere Technologies, Jena, Germany) was used for DNA microarray analysis on 10 representative isolates of the different MLVF patterns of ST22-IVa at the National Center for Antimicrobials and Infection Control, Statens Serum Institut, Copenhagen, Denmark. This kit allows for detection of 334 *S. aureus* gene sequences, including species-specific, antimicrobial resistance, and virulence genes as well as typing markers. The DNA microarray protocol was performed according to the manufacturer’s instructions. Data generated by the *S. aureus* genotyping kit arrays were analyzed using the Arraymate software (Alere Technologies), which assigns MRSA isolates to STs and/or clonal complexes (CCs) by comparing each isolate’s profile to those of a collection of previously characterized strains in the Arraymate database [13]. Fifteen representative strains of all the different MLVF patterns were also analyzed by multilocus sequence typing (MLST). The MLST allelic profiles and sequence types were assigned by submission to the *S. aureus* MLST database (www.mlst.net). Additionally, s*pa* typing was carried out, as previously described, on eight ST22-MRSA-IVa isolates representative of the different MLVF patterns [14]. The *spa* type was determined using the Ridom StaphType software (http://www.ridom.de/staphtype/).

### Statistical Analysis

Analysis of the variance (ANOVA) was carried out for comparing mean values differences between the three year-periods of the study. The Bonferroni pairwise procedure was adopted as post-hoc test. Correlation between the mean WCP values and the number of incident MRSA colonizations in the immediately following week was analysed using least-square linear regression.

The characteristics of the neonates with and without MRSA colonization as well as neonates with colonization with ST22-IVa and other MRSA strains were compared by calculating the means (standard deviation, SD) and frequencies. The significance of differences was assessed by one-way ANOVA test or Kruskall-Wallis, when appropriate, or by the chi-square test or the Fisher’s exact test, respectively. All reported P values were two-sided and p<0.05 was considered significant. Statistical analysis was performed by using EpiInfo (version 7; CDC, Atlanta, GA, US) and STATA MP, v. 11.0.

## Results

During the study period, 722 infants fulfilling the inclusion criteria were admitted to the NICU and enrolled into the study. Their main characteristics are summarized in Table 1.

**Table 1:** Characteristics of the patients at admission to the NICU, June 2009–June 2012, Palermo, Italy.

Study population	No. infants (%)
Gender (male)	409 (66.6)
Birth weight, g*	
≤1000	25 (3.5)
1001–1500	37 (5.2)
1501–2000	92 (12.8)
2001–2500	133 (18.5)
>2500	430 (60.0)
Gestational age, wk†	
24–29	35 (4.9)
30–36	237 (33.2)
>36	442 (61.9)
Inborn	428 (59.3)
Age at admission >24 h	108 (14.9)
Twin birth	104 (14.4)
Cesarean delivery	490 (67.9)
Apgar score at 5 min <8	88 (12.2)
Malformation	138 (19.1)

*information about birth weight was available for 717 out of 722 infants.

† information about gestational age was available for 714 out of 722 infants.

The annual outborn proportion of admissions raised from 30.8% to 32.9% and to 36.3%, although the increase was not statistically significant (*P* 0.09). Meanwhile, gestational age proved to gradually decrease from a mean of 37.0 weeks (median 38.0 weeks, interquartile range [IQR] 36.0–39.5 weeks) in the first year to 36.6 weeks (median 37.0 weeks, IQR 35.5–39.1 weeks) in the second year and to 36.2 weeks (median 37.0 weeks, IQR 34.5–39.0 weeks) in the third year (ANOVA, F = 7.69; Bonferroni test: first and second year, *P* 0.85; first and third year, *P*<0.001; second and third year, *P* 0.02).

The average length of stay was 18.9 days (median 11 days, IQR 7–22 days). Over the study period the median length significantly increased up to 14 days, IQR 8–26 days, in the third year from 9, IQR 7–22 days, in the first year and 10, IQR 7.5–18.5 days, in the second year (ANOVA, F = 3.61; Bonferroni test: first and second year, *P* 0.91; first and third year, *P* 0.02; second and third year, *P* 0.02). The mean number of patient-days by week increased also over the study period from 65.1±17.1 during the first year to 68.0±14.4 during the second year and 82.1±16.1 during the third year (ANOVA, F = 16.96; Bonferroni test: first and second year, *P* 0.89; first and third year, *P*<0.001; second and third year, *P*<0.001) (Table 2).

**Table 2:** Annual prevalence of MRSA in NICU, June 2009–June 2012, Palermo, Italy.

Year	No. patients	Patient-days	Mean number of patient-days by week	*P*	No. patients with MRSA	Patient-days with MRSA	Mean number of patient-days with MRSA by week	*P*	Mean weekly colonization pressure (WCP)*	*P*
1^st^ (2009–2010)	257	4356	65.1±17.1		88	643	12.4±8.4		19.1±10.7	
2^nd^ (2010–2011)	224	3864	68.0±14.4	<0.001	34	483	9.3±6.7	0.03	13.4±9.6	0.04
3^rd^ (2011–2012)	241	4962	82.1±16.1		65	729	13.8±10.9		16.8±13.7	

*number of MRSA patient-days in the week/total number of patient-days in the same week, expressed as percentage of patient-days.

Three hundred eighty (52.7%) infants received some antibiotic treatment during the NICU stay. Ampicillin-sulbactam and gentamicin were the most commonly used antimicrobials, being administered to 339 (89.2%) out of these patients for a mean of 3.3 days (median 3 days, IQR 1–5 days). A total of 2028 nasal swabs were cultured from the infants under study, with a mean of 2.8 specimens per infant (range 1–25). Overall, 187 (25.9%) out of 722 subjects tested positive for MRSA. The mean interval of time between admission and the first positive swab was 13.3 days (median 7 days, IQR 5–16 days).

Table 2 summarizes the number of colonized MRSA patients and the mean number of MRSA patient-days by year. This latter figure showed a value of 12.4±8.4 in the first year which declined to 9.3±6.7 in the second year to climb again to 13.8±10.9 during the third year of study (ANOVA, F = 3.58; Bonferroni test: 1st and 2nd year, *P* 0.23; 1st and 3rd year, *P* 0.98; 2nd and 3rd year, *P* 0.03). Consistently, the annual incidence density of acquisition of MRSA over the three-year period ranged from a maximum of 20.2 cases for 1000 patient-days during the first year to a minimum of 8.8 cases in the second one to raise again up to 13.1 cases during the third year. Incidence of clinical infections did not significantly changed over the study period: it was 5.2 per 1000 patient-days in the first year, 6.5 per 1000 patient-days in the second year and 4.9 per 1000 patient-days in the last year, *P* 0.48.

**Figure 1:**
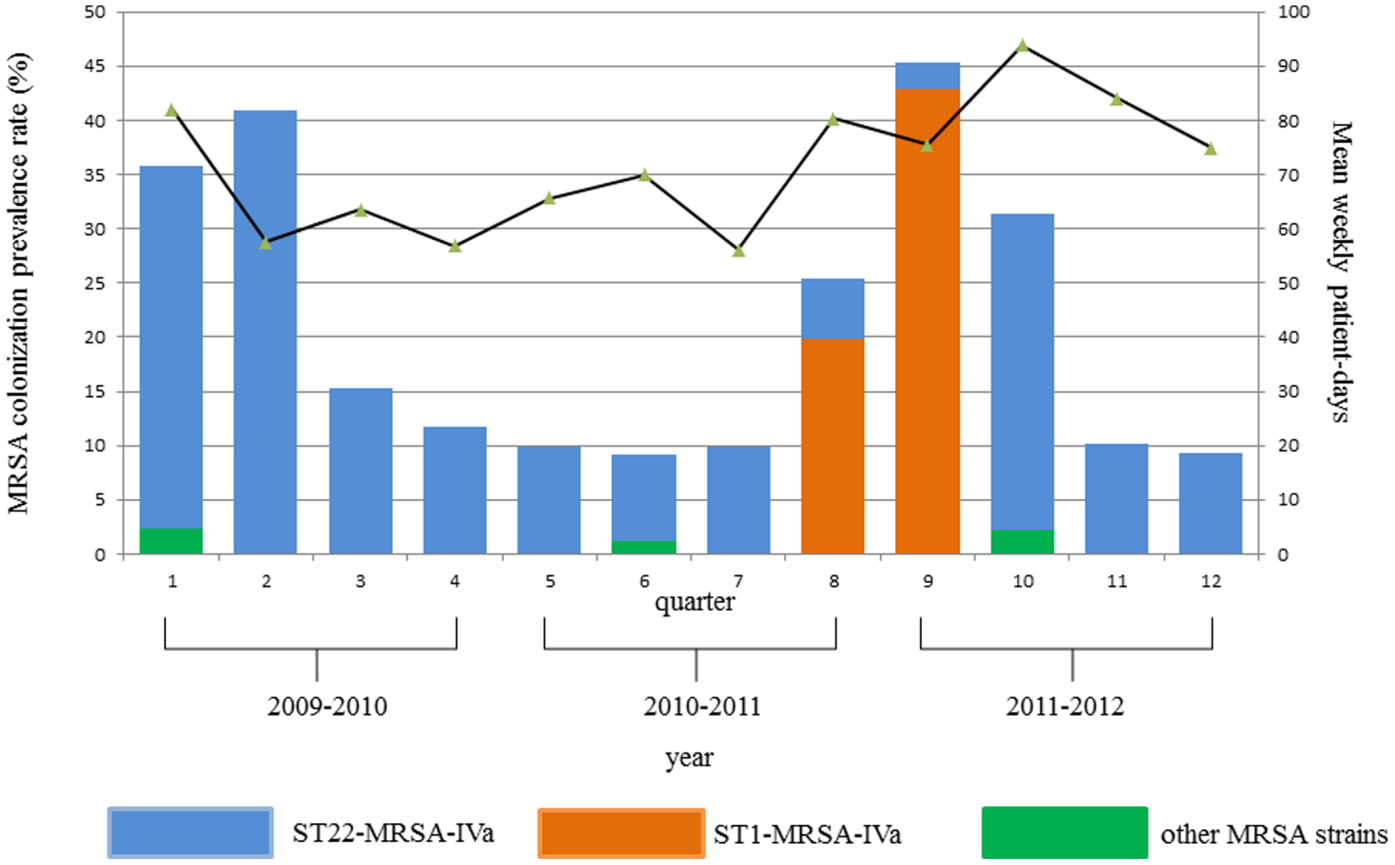
Prevalence of colonization by the different MRSA strains and mean weekly patient-days by quarter in the NICU under study, June 2009–June 2012, Palermo, Italy.

As Figure 1 illustrates, following the high prevalence rates recorded in the first semester and the implementation of targeted control strategies, the MRSA colonization prevalence decreased to about 10%. However, a new dramatic rise occurred in the 8th to 10th quarters concurrently with the entry into the NICU of ST1-MRSA-IVa and, soon afterwards, with a period of substantial overcrowding. Indeed, through the entire period of observation a mean of 70–95% beds had been used continuously, whereas in the 10th quarter the bed occupancy rate exceeded the standard of 16 beds by a mean of 15% in eight out of 13 weeks.

WCP ranged from 0% to 60.9% (mean 16.4±11.6) over the study period. The changes over time are summarized in Table 2. Significant differences were observed between the first two years (ANOVA, F = 3.25; Bonferroni test: 1st and 2nd year, *P* 0.04; 1st and 3rd year, *P* 0.76; 2nd and 3rd year, *P* 0.48). WCP significantly correlated with the number of MRSA acquisitions in the following week (correlation coefficient 0.77, *P* 0.009).

### Characterization of MRSA Isolates

Overall, seven sequence types (STs) were identified, with ST22-IVa being the predominant (166 out of 187, 88.8%). SCC*mec* type IVa was identified in all MRSA isolates (Table 3). ST22-IVa isolates were further subdivided by MLVF into eight different subtypes. The largest number of isolates belonged to the MLVF subtype ST22-A, which was detected through the entire period of the study. Conversely, the detection of isolates belonging to the two additional larger groups, i.e. ST22-IVa subtype 22-C and ST1-IVa, was concentrated within more restricted intervals of time (Figure 1 and Table 3).

**Table 3:** Molecular characteristics, antibacterial drug resistance pattern and time of isolation of isolates belonging to the different clones.

Sequence type	SCC-*mec* type	MLVF	No. of isolates		Antimicrobial susceptibility pattern										Quarter
				Cip	Cli	Ery	Gen	Lin	Rif	Sxt	Tec	Tet	Tob	Va	
22	IVa	22-A	67	S	S	S	S	S	S	S	S	S	S	S	1–12
22	IVa	22-A	67	S	S	S	S	S	S	S	S	R	S	S	1–12
22	IVa	22-B	4	S	S	S	S	S	S	S	S	S	S	S	2,4
22	IVa	22-C	17	S	S	S	S	S	S	S	S	R	S	S	2,3
22	IVa	22-D	2	S	S	S	S	S	S	S	S	R	S	S	8
22	IVa	22-E	1	S	S	S	S	S	S	S	S	R	S	S	8
22	IVa	22-F	1	S	S	S	S	S	S	S	S	R	S	S	9
22	IVa	22-F	1	S	S	S	S	S	S	S	S	S	S	S	9
22	IVa	22-H	4	S	S	S	S	S	S	S	S	S	S	S	10
22	IVa	22-I	2	S	S	S	S	S	S	S	S	S	S	S	11
8	IVa	8-A	1	R	S	R	S	S	S	S	S	S	S	S	5
1	IVa	1-A	15	S	S*	R	R	S	S	S	S	S	S	S	8,9
7	IVa	7-A	1	S	S	S	S	S	S	S	S	R	S	S	10
20	IVa	20-A	1	S	S	R	S	S	S	S	S	R	S	S	1
45	IVa	45-A, 45-B	2	S	S	S	R	S	S	S	S	S	S	S	5,9
97	IVa	97-A	1	S	S	S	R	S	S	S	S	R	S	S	5

SCC-*mec*, staphylococcal cassette chromosome *mec*; MLVF, multilocus variable number tandem repeat fingerprinting; S, susceptible; R, resistant.

Cip, ciprofloxacin; Cli, clindamycin; Ery, erythromycin; Gen, gentamicin; Lin, linezolid; Rif, rifampicin; Sxt, sulfamethoxazole-trimethoprim; Tec, teicoplanin; Tet, tetracycline; Tob, tobramycin; Va, vancomycin.

*Inducible clindamycin-resistant phenotype.

As Table 3 illustrates, the MRSA isolates were fully susceptible or, alternatively, resistant to a limited number of non-β lactam antibiotics. In particular, none was resistant to sulfamethoxazole-trimethoprim, tobramycin, linezolid, rifampicin, vancomycin and teicoplanin. Only the ST1-IVa isolates showed an inducible clindamycin resistant phenotype.

No isolate was found to carry the PVL gene sequence. The DNA microarray profiling assigned the ST22 MRSA isolates to the CC22-MRSA-IV tst1+, UK-EMRSA-15/ “Middle Eastern Variant”. These isolates were also negative for the *sec* and *sel* genes. *spa* typing assigned them with type t223.

The isolates belonging to STs other than ST22 tested all negative for *tst*1.

### Characteristics of MRSA Colonized and Non Colonized Infants

Demographic, clinical and healthcare related parameters of MRSA colonized and non colonized infants are summarized in Table 4. Compared with no colonization, the infants with MRSA colonization were significantly more inborn than outborn. Conversely, male gender and admission to NICU ≥24 h after birth were negatively correlated with MRSA colonization (Table 4). During the NICU stay, use of invasive devices was not significantly different between the two groups of infants, except for insertion of central venous catheters (CVCs) which was significantly less frequent among those colonized with MRSA. Administration of ampicillin-sulbactam plus gentamicin and, more generally, of systemic antibacterial therapy, was significantly more frequent among the non colonized neonates. Length of stay was significantly longer for the colonized infants (Table 4). Neither sepsis [colonized versus non colonized, 24 (12.8%) versus 49 (9.2%), *P* 0.09] nor in-hospital death [colonized versus non colonized, 5 (2.7%) versus 8 (1.5%), *P* 0.30] were significantly associated with MRSA colonization among the infants under study.

**Table 4:** Comparison between characteristics at admission and during NICU stay of MRSA colonized and non colonized infants, June 2009–June 2012, Palermo, Italy.

Variable	Colonized (n = 187)	Noncolonized (n = 535)	*P*
At admission			
Male gender, n (%)	88 (47.1)	321 (60.0)	0.001
Twin birth, n (%)	31 (16.6)	73 (13.6)	0.15
Malformation, n (%)	40 (21.4)	98 (18.3)	0.18
Inborn, n (%)	135 (72.2)	293 (54.8)	<0.001
Birth through vaginal delivery, n (%)	52 (27.8)	158 (29.5)	0.29
Admission to NICU ≥24 h after birth, n (%)	16 (8.6)	92 (17.2)	0.001
Apgar score at 5 min ≥8, n (%)	163 (87.2)	461 (86.2)	0.43
Gestational age, mean (SD), wk	36.4 (3.5)	36.7 (3.3)	0.23
Birth weight, mean (SD), g	2568 (867)	2673 (760)	0.12
During stay			
Central venous access device, n (%)	51 (27.3)	192 (35.9)	0.01
Endotracheal tube, n (%)	37 (19.9)	114 (21.3)	0.33
Nasogastric tube, n (%)	71 (38.0)	201 (37.6)	0.47
nCPAP, n (%)	39 (20.9)	102 (19.1)	0.30
Parenteral nutrition, n (%)	80 (42.8)	270 (50.5)	0.07
Surgical procedure, n (%)	8 (4.3)	38 (7.1)	0.10
Formula feeding, n (%)	181 (96.8)	496 (92.7)	0.07
Breast milk feeding, n (%)	97 (51.9)	275 (51.4)	0.46
Length of stay, mean (SD), d	25.3 (30.9)	16.6 (16.7)	0.02
Ampicillin-sulbactam plus gentamicin, n (%)	73 (39.0)	266 (49.7)	0.005
Ampicillin-sulbactam plus gentamicin, mean (SD), d	4.8 (7.3)	5.0 (6.3)	0.36
Systemic antibacterial therapy, n (%)	83 (44.4)	297 (55.5)	0.004
Systemic antibacterial therapy, mean (SD), d	7.1 (14.2)	5.8 (9.1)	0.07

SD, standard deviation; nCPAP, nasal continuous positive airway pressure.

When comparing the MRSA colonized neonates based upon the MRSA strain, i.e. ST22-MRSA-IVa versus other MRSA strains, the infants colonized by these latter ones were significantly more likely to be admitted to NICU ≥24 h after birth and their gestational age was significantly lower than those colonized with the ST22 strain. Moreover, they were more frequently submitted to surgery and exposed to invasive devices (Table 5). Antibacterial treatment was also significantly more frequent and protracted in this group of patients. (Table 5).

**Table 5:** Comparison between infants colonized by ST22-MRSA-IVa and those colonized by other genotypes, June 2009–June 2012, Palermo, Italy.

Variable	Infants with ST22-MRSA-IVacolonization (n = 166)	Infants with other MRSAclones colonization (n = 21)	*P*
At admission			
Male gender, n (%)	79 (47.6)	9 (42.9)	0.35
Twin birth, n (%)	28 (17.7)	3 (14.3)	0.37
Malformation, n (%)	98 (18.3)	40 (21.4)	0.18
Inborn, n (%)	35 (21.5)	5 (25.0)	0.35
Birth through vaginal delivery, n (%)	45/27.8)	7 (33.3)	0.30
Admission to NICU ≥24 h after birth, n (%)	11 (6.6)	5 (23.8)	0.02
Apgar score at 5 min ≥8, n (%)	146 (90.1)	17 (81.0)	0.18
Gestational age, mean (SD), wk	36.6 (3.3)	34.1 (4.1)	0.001
Birth weight, mean (SD), g	2604 (846)	2284 (996)	0.11
During stay			
Surgical procedure, n (%)	5 (3.1)	3 (15.0)	0.04
Central venous access device, n (%)	39 (23.5)	12 (57.1)	0.001
Endotracheal tube, n (%)	29 (17.5)	8 (38.1)	0.03
Nasogastric tube, n (%)	60 (36.1)	11 (52.4)	0.08
nCPAP, n (%)	29 (17.5)	10 (47.6)	0.003
Parenteral nutrition, n (%)	64 (38.6)	16 (76.2)	<0.001
Length of stay, mean (SD), d	24.6 (31.1)	30.8 (28.9)	0.38
Ampicillin-sulbactam plus gentamicin, n (%)	61 (36.7)	12 (57.1)	0.05
Ampicillin-sulbactam plus gentamicin, mean (SD), d	4.0 (7.0)	7.8 (9.1)	0.03
Systemic antibacterial therapy, n (%)	69 (41.6)	14 (66.7)	0.02
Systemic antibacterial therapy, mean (SD), d	6.3 (13.0)	14.0 (20.7)	0.02

SD, standard deviation; nCPAP, nasal continuous positive airway pressure.

Sepsis [ST22 versus other MRSA colonized infants, 19 (11.4%) versus 6 (28.6%), *P*0.04] was significantly associated with colonization by other MRSA strains than ST22. Conversely, no association was apparent with in-hospital death [ST22 versus other MRSA strains, 4 (2.4%) versus 1 (4.8%), *P* 0.45].

## Discussion

Neonates are highly susceptible to MRSA colonization. Colonization and infection in NICU have been reported in many countries and have been attributed to HA- or, with an increasing frequency, to CA-MRSA strains [2]. MRSA can enter the NICU via the family members of the neonates, the HCWs or the outborn patients [15]–[18]. Blurring of boundaries between healthcare settings and community is increasingly hindering an accurate discrimination of HA- and CA-MRSA strains, when relying on conventional epidemiology tools only [19].

Since 2000, in most European countries the epidemiology of MRSA is evolving with a decreasing prevalence of multidrug resistant MRSA clones and, conversely an increasing frequency of non β-lactam susceptible clones. Among these latter clones, ST22-IV (EMRSA-15] has a prominent role being one of the three most frequent MRSA clones in seven out of 15 European countries [20]–[21]. It is characteristically ciprofloxacin resistant and gentamicin susceptible, rarely produces PVL, harbours SCC*mec* subtype IVh and has *spa* type t022 or t032 or variants [21]. Fluoroquinolone resistance appears to have played a key role in initiating pandemic spread. However, the EMRSA clone ST22-IV has some further properties which could explain its attitude to replace previously well-established hospital associated strains, such as an increasing biofilm production over time, the ability to maintain its growth kinetics in presence of competitor clones and a more frequent association with persistent bacteremia compared to other HA clones [21], [22]. A correlation between gentamicin susceptibility and the enhanced ability of MRSA to survive and spread in healthcare environments has been also reported [23]. A reason of further concern is the tendency of ST22-IV to escape from the healthcare environment to the community, which has been described in highly endemic settings [19], [24].

Our epidemiological landscape appears to be consistent with this evolving trend. Indeed, a predominance of ST22-IV among the non multiresistant isolates identified during 2009 in four hospitals in Palermo, Italy, has been previously highlighted [25]. More recently, ST22-IVa was identified in eight out of 10 MRSA carrying healthy children among a sample of 500 attending the municipal child care centers of the same city [26]. However, our ST22 isolates are distinctly different from the European pandemic isolates. They, indeed, were identified as belonging to the *tst*1 positive, UK-EMRSA-15/ “Middle Eastern Variant” and were also ciprofloxacin susceptible and *sec* and *sel* gene negative [27]. Moreover, they had a distinct *spa* type t223 and carried SCC*mec* subtype a. Our ST22 variant seems to be identical to the “Gaza strain” recently described by Biber A. et al. [28] as widely spreading in community in the Gaza strip. Isolates with similar characteristics have been also reported in Saudi Arabia, Abu Dhabi and Egypt [27], [29]. Moreover, isolation of *tst1-*positive CC22-IV isolates has been described in England from patients of apparent Middle Eastern origin and for the first time from a sporadic case in USA [29]. Our data suggest that this clone is likely more widely diffuse than it has been estimated and is spreading in the Mediterranean area. Further investigations are necessary to assess if this strain is a true HA clone, such as the classic EMRSA-15, or, alternatively, a CA clone that might have entered the NICU via the HCWs, the parents or the newborns. According with Biber et al. [28], the relationship with local ST22-MSSA strains could help answering this question. As previously reported, MLVF was able to timely identify new circulating strains into the NICU and to sub-cluster isolates belonging to the same ST and *spa* type and endemically present in the ward [30].

At the beginning of our surveillance activity, the NICU proved to be high-endemic for MRSA. A bundle of infection control procedures was soon started which proved to be able to decrease the colonization rate from more than 40% to approximately 10% until the seventh quarter of the study period. The following sudden rise in MRSA prevalence was attributed with the emergence and spread of the CA-MRSA strain ST1-IVa followed by an epidemic cluster of colonization cases by ST22-IVa [31]. Indeed, strengthening of the infection control procedures due to the alarm generated by the entry in NICU of the gentamicin resistant ST1-IVa strain proved to be effective in gaining eradication of this strain, but unable to prevent a subsequent temporary recrudescence of ST22-IV colonization. Many concurrent factors likely contributed to this adverse outcome: the third year of study was, indeed, characterized by a higher mean number of weekly patient-days, a lower mean gestational age, a longer average length of stay and a higher rate of outborn infants than the previous two years. WCP also, following a significant decrease during the second year, came back at the same value of the first year. Moreover, just during the second quarter of the third year, NICU was significantly crowded with an average bed occupancy rate substantially exceeding the standard level. The concurrent emergence and spread in NICU of a strain of carbapenemase producing *Klebsiella pneumoniae* could have had a further adverse impact on MRSA ST22-IVa transmission by distracting most infection control efforts towards eradication of that organism [32]. Overcrowding is universally agreed as a major driving factor of cross-transmission, mainly because of failure in adequately performing hand hygiene procedures [2], [5]. On the other hand, a high colonization pressure, i.e. >50%, can substantially hamper effectiveness of infection control measures, including active surveillance, and can require more aggressive control strategies [33]. However, the rigorous application of infection control policies and procedures was again successful in bringing MRSA colonization prevalence rate back to about 10%.

In our experience, MRSA colonized and non colonized infants did not differ except for a few characteristics. The inborn infants were more likely to be MRSA colonized. This might reflect a differential admission bias of parturients since the highest risk pregnancies are more likely to be delivered in a maternity ward with an annexed specialized NICU. On the contrary, non colonized infants were more likely to be of male gender and later admitted after birth than the colonized. Administration of a systemic antibacterial drug therapy looked like to be more frequent in non colonized infants, as a probable consequence of the gentamicin susceptibility of the predominant MRSA clone. Literature is not quite unanimous about risk factors for colonization by MRSA strains in NICU patients and an association between MRSA acquisition and the most reputed determinants, such as gestational age, birth weight, presence of underlying disease, CVCs or previous treatment with antibiotics is not always reported [4]. Moreover, colonization pressure in high endemic settings is expected to accelerate MRSA acquisition regardless of the risk factors and, consequently, to level out the differences between colonized and non colonized infants. However, many variables commonly associated with nosocomial acquisition of MRSA, such as lower gestational age, more frequent use of invasive devices and administration of antibiotics, proved to be more likely associated with infants colonized by MRSA strains other than ST22-IV. Moreover, while no significant differences were detected in frequency of sepsis and in-hospital death between MRSA colonized and non colonized infants, infants colonized with non ST22 strains were significantly more likely to develop sepsis. Of interest, the majority of the non ST22 strains were ST1, a typical CA-MRSA clone, which has been responsible for an epidemic spread following its importation into the NICU via an infected infant from another healthcare facility [31].

MRSA spread in NICU has been reported to be difficult to control [34]–[38], due also to its possible (re)introduction from different sources over time. Only implementation of aggressive infection control measures, frequently combined with mupirocin treatment of infants and HCWs and, more recently, chlorhexidine gluconate body wash of colonized infants, has proved to be successful in controlling some outbreaks [35], [39]. However, poor success of mupirocin decolonization has also been reported, mainly in association with MRSA colonization of body sites other than nares [40]. Moreover, besides the issue of the possible selection of mupirocin resistant strains, there are safety concerns regarding usage of decolonizing agents in preterm infants and newborns [40]. A further issue of concern is the possible selection of non susceptible Gram negatives which may colonize patients after chlorexidine application or, more insidiously, contaminate disinfectant products [41]. More generally, data supporting cost-effectiveness of NICU specific infection control strategies are limited and further studies are warranted.

During the three-year period of surveillance, we registered a considerable decrease of the prevalence of MRSA colonization in NICU, from 41% to 9%, a favourable trend which was transiently interrupted by the introduction of the ST1-IVa strain and a recrudescence of colonization by ST22-IVa, as above discussed. A multifaceted infection control intervention was implemented and is still in place, except for screening and decolonization of HCWs. Since all the measures were undertaken simultaneously, it is impossible to define which of the measures was the most effective. However, they were unable to definitively eradicate MRSA from the NICU. This partial failure can supposedly result from many concurrent factors, such as periodic overcrowding and relative understaffing, the peculiar case-mix of patients in our NICU, possible recurrent introductions of MRSA from community through parents or HCWs, failure to strictly adhere to recommended practices for long periods and the relative ineffectiveness of routine procedures in controlling strains with a high fitness in the hospital setting, such as ST22 [22], [23]. The peculiar susceptibility of newborns and their frequent contacts with possible carrier adults further contribute to the MRSA persistence.

Our study has some limitations. It was performed in a single NICU in an university affiliated general hospital, which is also a reference center for genetic diseases. This can limit generalizability of the results. Screening of newborns at admission was only performed during the first six months of the study, as well as screening of HCWs. So, timing of MRSA acquisition in NICU was not traceable and assessment of risk factors was not feasible. Moreover, screening of parents and family members was not performed at all. In summary, our MRSA surveillance program did not include active efforts aimed to identify sources of MRSA other than the infants. Along with the limited epidemiological data about community MRSA carriage in our geographic area, some gaps in our surveillance system do not allow understanding the dynamics of MRSA introduction in the NICU. Moreover, continuous quantitative data regarding nurse-to-patient ratio and adherence of staff to infection control procedures, such as hand washing, were not collected.

## Conclusions

This is the first study to provide prospective baseline data on the prevalence of MRSA in our NICU. Furthermore, the molecular characterization has shown to be valuable to detect the emergence and spread of the various circulating clones. However, surveillance data from other hospitals who admit patients to our NICU or from the community could be helpful in elucidating most probable routes of MRSA entry into the NICU.

The NICU is a complex ecosystem, where interactions between pre-admission factors, both maternal and infant-related, MRSA strain factors and healthcare setting factors, including patient management and contacts with the staff, are a continuous challenge for infection control. In our experience, an endemic presence of ST22-MRSA-IVa was accompanied by the introduction of multiple MRSA strains which sporadically or epidemically colonized NICU infants. Unlike clinical cultures which would have seriously underestimated affected neonates, active surveillance contributed to obtain a reliable picture of the MRSA dissemination in NICU. Risk factors for MRSA colonization in NICU infants as well as impact of organizational and structural issues deserve further studies in different settings with various levels of endemicity and different patterns of circulating strains.
